# Proposing preoperative neutrophil percentage-to-albumin ratio as an effective prognostic factor for postoperative prognosis of distal cholangiocarcinoma: a retrospective cohort study

**DOI:** 10.3389/fmed.2025.1725834

**Published:** 2026-01-12

**Authors:** Han-xuan Wang, Zu-yu Wang, Shao-cheng Lyu, Jin-can Huang, Qiang He, Ren Lang, Tao Jiang

**Affiliations:** Division of Hepatobiliary and Pancreaticosplenic Surgery, Department of General Surgery, Beijing Chao-Yang Hospital, Capital Medical University, Beijing, China

**Keywords:** biomarker, distal cholangiocarcinoma, neutrophil percentage-to-albumin ratio, postoperative prognosis, prognostic value

## Abstract

**Background:**

The neutrophil percentage-to-albumin ratio (NPAR) is a novel inflammatory-nutritional biomarker that has prognostic value in various cancers. This study aimed to evaluate the predictive value of preoperative NPAR for postoperative prognosis in patients with distal cholangiocarcinoma (dCCA).

**Methods:**

We retrospectively analyzed dCCA patients who underwent radical surgery between January 2011 and December 2023. Independent risk factors were identified using univariate and multivariate analysis. The prognostic performance of NPAR was assessed using receiver operating characteristic (ROC) curve analysis, and the optimal cutoff value was determined. Survival outcomes were compared using the log-rank test, and propensity score matching (PSM) was applied to adjust for confounders. Predictive models were developed using multiple machine-learning algorithms.

**Results:**

Among 192 included patients, NPAR demonstrated an area under the ROC curve of 0.702 inpredicting postoperative survival, with an optimal cutoff value of 1.735. Multivariate analysis confirmed that elevated preoperative NPAR was an independent risk factor for both disease-free survival (DFS) and overall survival (OS). Patients with NPAR > 1.735 had significantly worse postoperative outcomes. Subgroup analysis indicated that NPAR had stronger predictive value in patients with tumor invasion depth > 12 mm, but without portal vein invasion or lymph node metastasis. Machine-learning models incorporating NPAR improved the prediction of postoperative prognosis in dCCA.

**Conclusion:**

Elevated preoperative NPAR (>1.735) is an independent risk factor for postoperative DFS and OS in dCCA patients and may serve as a potential prognostic index.

## 1 Introduction

Cholangiocarcinoma is a relatively rare malignancy with significantly different incidence worldwide. Despite a low incidence rate of just 0.35 per 100,000 in developed countries, cholangiocarcinoma poses a higher disease burden in China, where the overall incidence is up to 40 times greater (1, 2). Distal cholangiocarcinoma (dCCA) is a kind of malignancy located below the conjunction of gallbladder duct and common hepatic duct (3). Radical pancreatoduodenectomy remains the most important treatment for dCCA, but the postoperative prognosis of dCCA is still unsatisfying (4). Early prediction of postoperative prognosis in dCCA patients is critical for guiding subsequent therapy and improved overall prognosis.

Inflammation is closely correlated with the development and progression of malignancy, a relationship that holds true for cholangiocarcinoma as well. Factors contributing to local chronic inflammation and systemic chronic inflammation factors have been identified as risk factors for the development of cholangiocarcinoma. Moreover, inflammation has been shown to enhance the progression and metastasis of cholangiocarcinoma (5, 6). Neutrophils, an important component of innate immunity, have been confirmed to infiltrate the tumor microenvironment, where they exacerbate progression of cholangiocarcinoma and substantially reduce the overall survival rates of dCCA patients (7, 8). Various studies have confirmed the clinical value of neutrophil-related indices in predicting postoperative prognosis of dCCA (9–11). Given that malnutrition is a known risk factor for poor prognosis of dCCA, and that serum albumin is a key laboratory indicator of nutritional status, researchers also evaluated and confirmed the prognostic value of the albumin-related indices including prognostic nutritional index (PNI), C-reactive protein-to-albumin ratio (CAR) and C-reactive protein-albumin-lymphocyte ratio (CALLY) for dCCA patients (12–15).

Neutrophil percentage-to-albumin ratio (NPAR) is a newly proposed inflammatory index including both neutrophil ratio and serum albumin. It was first proposed by Cui et al. in 2019 to predict the risk of in-hospital death in patients with ST-elevation myocardial infarction. It predictive value may be attributed to its ability to reflect the severity of inflammation and malnutrition status according to researchers (16). Recently, the prognostic ability of NPAR has also been extended to colorectal and bladder cancer according to researches (17, 18). However, the prognostic significance of NPAR in dCCA has not been investigated so far. Considering that the inflammation and nutritional status are closely correlated with the prognosis of dCCA, we speculated that NPAR may exert predictive value for postoperative prognosis in dCCA patients. Current study aimed to analyze dCCA patients who underwent radical surgery in a retrospective cohort and explore the potential value of NPAR in predicting postoperative prognosis of dCCA.

## 2 Methods

### 2.1 Patient selection

This study retrospectively analyzed the data of dCCA patients who were admitted to department of hepatobiliary and pancreaticosplenic surgery, Beijing Chaoyang Hospital from January 2011 to December 2023 and received surgical treatment. The patients were selected according to the following inclusion and exclusion criteria.

### 2.2 Inclusion criteria

(1) Patients suspected of dCCA and underwent surgical treatment in our department from January 2011 to December 2023; (2) Preoperative evaluation confirmed no surgical contraindications; (3) Achieved en-bloc resection intraoperatively; (4) Postoperative pathology confirmed the diagnosis of dCCA; (5) Complete clinical and follow-up data.

### 2.3 Exclusion criteria

(1) Acute cholangitis or pancreatitis due to biliary obstruction or biliary drainage before surgery; (2) Combined other infectious diseases or hematologic malignancy before surgery; (3) Preoperative supplementation of albumin; (4) Suffered perioperative death.

Participant informed consent was waived due to the retrospective study design, and the acquisition of clinical data of patients was approved by the Ethics Committee of Beijing Chaoyang Hospital Affiliated to Capital Medical University (Granted number: 2024-D.-511).

### 2.4 Patients grouping

The last neutrophil percentage and serum albumin results within 1 week before surgery were included for analysis, and NPAR was calculated by the formulation of neutrophil percentage × 100/serum albumin., The optimal cut-off value were calculated according to receiver operator characteristics curve (ROC) between NPAR and postoperative survival of patients. Included patients were further categorized into low and high NPAR group with the optimal cut-off value for further analysis.

### 2.5 Index analysis and follow-up

Preoperative, intraoperative and postoperative data of included patients were obtained from the medical record system of our hospital and were compared within groups. Follow-up was conducted via telephone and outpatient visits. Patients were first followed up at 1 month and 3 months after surgery. Then they were followed up every 3 months in postoperative 2 years and every 6 months afterward until mortality. The primary endpoint of this research was postoperative mortality and the secondary endpoint was tumor recurrence. The results of blood examination (blood routine, blood biochemistry, tumor markers) and imaging examination (abdominal enhanced CT, lung CT), subsequent treatment schedule, tumor recurrence and survival condition were obtained during follow-up.

### 2.6 Statistical analysis

Non-normally distributed continuous data were expressed as the median (interquartile range) and were compared with rank sum tests. Chi-square test was used to compare categorical data, and Fisher's test was used when sample size was no more than 40 or the theoretical frequencies less than 1. For missing data, non-normally distributed continuous variables with a missing rate no more than 5% were imputed using the median, while those continuous variables with a missing rate exceeding 5% were excluded from further analysis. Patients with missing categorical variables or with missing follow-up data were excluded from the analysis. ROC curve was used to evaluate the prognostic value of indexes and determine the optimal cut-off value of NPAR. Multicollinearity among NPAR, absolute neutrophil count, neutrophil percentage and albumin was assessed by calculating the variance inflation factor (VIF) in a linear regression model, and VIF <5 indicated severe multicollinearity. Variables with statistical difference in intergroup comparison were adjusted by propensity score matching (PSM) using nearest neighbor matching in a 1:1 ratio without replacement. The Caliper width was set as 0.2. Restricted cubic splines (RCS) analysis was applied to evaluate the correlation between the variation of NPAR and hazard risk of postoperative prognosis, which was conduction by “rcssci” package. Kaplan–Meier method was utilized to calculate survival curves, Log-rank test was used to compare the differences in survival rates between groups. Least Absolute Shrinkage and Selection Operator (LASSO) regression was applied to screen out potential risk factors using “glmnet” package, and Cox regression model was applied for multivariate analysis to select independent risk factors. Included patients were randomly allocated into a training set and a validation set in a 7:3 ratio. Prediction models were established, evaluated and visualized with “mime1” package, stepwise Cox regression and Ridge regression were performed with “survival” and “glmnet” package. All data are analyzed using SPSS (Version 26.0) and R Studio (Version R 4.5.0), with *P* < 0.05 considered statistically significant.

## 3 Results

### 3.1 General condition of included patients

A total of 192 patients were enrolled in this study, including 116 males and 76 females. The age of included patients was 65.0 (60.0, 73.0) years. Eight of patients who were suspected of portal vein system invasion received neoadjuvant chemotherapy. The treatment regimen including GC (Gemcitabine + Cisplatin, *n* = 6), GEMOX (Gemcitabine + Oxaliplatin, *n* = 2), and the cycle of neoadjuvant chemotherapy was 4.0 (2.0, 6.0). All enrolled patients underwent radical pancreaticoduodenectomy and additionally received vascular resection and reconstruction of portal vein system intraoperatively if they were detected of portal vein system invasion during surgery. The median operation time was 9.0 (7.0, 11.0) hours, and the median intraoperative blood loss was 500.0 (400.0, 700.0) ml. 63 patients (32.8%) developed complications after surgeries, among which 26 cases (13.5%) suffered from Clavien–Dindo grade III or higher complications. The median hospital stay after surgery was 22.0 (17.0, 32.0) days.

Postoperative pathological examination confirmed dCCA in all patients, including 26 cases (13.5%) of high-differentiated tumor, 100 cases (52.1%) of moderate-differentiated tumor and 66 cases (34.4%) of low-differentiated tumor. Portal vein system invasion and lymph node metastasis were observed in 26 patients (13.5%) and 84 patients (43.8%), respectively. Of the entire cohort, 183 patients (95.3%) achieved R0 resection, and the remaining 9 (4.7%) had R1 resection.

The follow-up of our research ended in August 2024, during which 96 patients (50.0%) received postoperative adjuvant chemotherapy. The median disease-free survival (DFS) time of included patients was 25 months, and the postoperative 1-year, 3-year and 5-year survival rates were 66.5%, 43.4% and 35.0%, respectively. The median overall survival (OS) time was 28 months, and the overall survival rates at 1, 3, and 5 years were 79.8%, 39.6%, and 31.6%, respectively.

### 3.2 Preoperative NPAR served as an independent risk factor for both postoperative tumor recurrence and overall survival in dCCA

**Figure 1:**
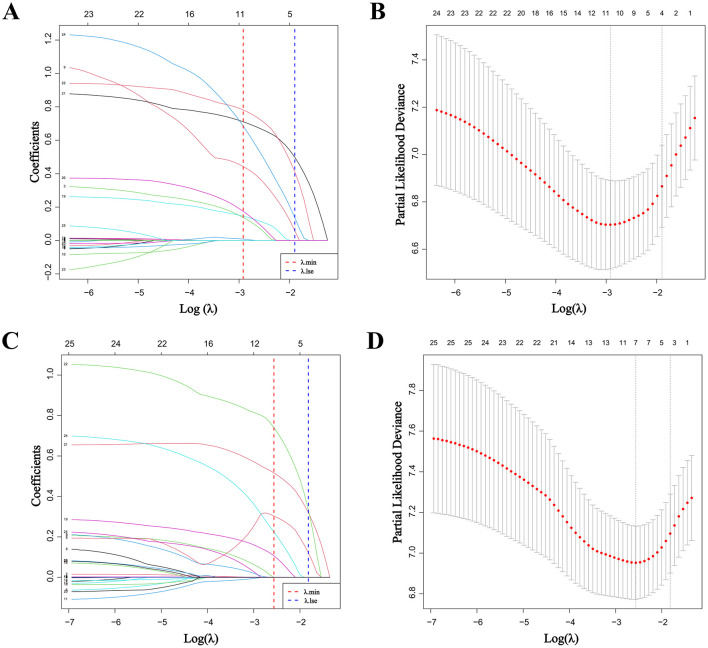
Result of LASSO regression: **(A)** coefficient path plot of LASSO regression identifying potential risk factors for postoperative tumor recurrence; **(B)** cross-validation curve of LASSO regression identifying potential risk factor for postoperative tumor recurrence; **(C)** coefficient path plot of LASSO regression identifying potential risk factors for postoperative overall survival; **(D)** cross-validation curve of LASSO regression identifying potential risk factor for postoperative overall survival (LASSO, least absolute shrinkage and selection operator).

To demonstrate the prognostic value of NPAR, we first performed LASSO regression to identify potential risk factors for postoperative tumor recurrence. Smoking, preoperative neutrophil count, preoperative NPAR, preoperative carbohydrate antigen 19-9 (CA19-9), intraoperative blood loss, tumor differentiation degree, tumor infiltration depth, lymph node metastasis, portal vein system invasion, and postoperative chemotherapy were potential risk factors for postoperative tumor recurrence in dCCA patients and were further included in multivariate Cox regression analysis (Figures 1A, B). The result showed that preoperative NPAR, tumor infiltration depth >12mm, lymph node metastasis, portal vein system invasion and postoperative chemotherapy were independent risk factors for postoperative tumor recurrence in dCCA patients (Table 1).

**Table 1:** Result of multivariate analysis of risk factors for post-operative tumor recurrence in patients with distal cholangiocarcinoma.

**Variables**	**RR**	**95%CI**	***P-*value**
Smoking	1.355	0.896–2.049	0.150
Preoperative neutrophil count (× 10^9^/L)	1.041	0.931–1.164	0.483
Preoperative NPAR	1.851	1.253–2.733	0.002
Preoperative CA19-9 (U/mL)	1.000	1.000–1.000	0.226
Intraoperative blood loss (mL)	1.000	1.000–1.000	0.129
Low tumor differentiation degree	1.247	0.829–1.874	0.290
Tumor infiltration > 12mm	3.444	1.585–7.482	0.002
Lymph node metastasis	2.273	1.513–3.414	<0.001
Portal vein invasion	2.489	1.474–4.204	0.001
Postoperative chemotherapy	0.674	0.456–0.998	0.049

RR, relative risk; CI, confidence interval; NPAR, neutrophil percentage-to-albumin ratio; CA19-9, carbohydrate antigen 19-9.

Moreover, we performed LASSO regression to screen out potential risk factors for postoperative overall survival for dCCA patients, and the result showed that preoperative NPAR, preoperative total bilirubin, preoperative CA19-9, tumor differentiation degree, tumor infiltration depth, lymph node metastasis, and portal vein system invasion were potential risk factors for postoperative overall survival and were included in multivariate Cox regression analysis (Figures 1C, D). The result showed that preoperative NPAR, preoperative CA19-9, lymph node metastasis and portal vein system invasion were independent risk factors for postoperative prognosis in dCCA patients. These results confirmed elevated preoperative NPAR level as an important risk factor for postoperative OS and DFS (Table 2).

**Table 2:** Result of multivariate analysis of risk factors for post-operative overall prognosis in patients with distal cholangiocarcinoma.

**Variables**	**RR**	**95%CI**	***P*-value**
Preoperative NPAR	1.461	1.042–2.048	0.028
Preoperative total bilirubin (μmol/L)	1.002	1.000–1.004	0.098
Preoperative CA19-9 (U/mL)	1.000	1.000–1.000	0.002
Low tumor differentiation degree	1.343	0.897–2.009	0.152
Tumor infiltration > 12mm	1.836	0.970–3.475	0.062
Lymph node metastasis	1.951	1.317–2.888	0.001
Portal vein invasion	2.789	1.689–4.606	<0.001

RR, relative risk; CI, confidence interval; NPAR, neutrophil percentage-to-albumin ratio; CA19-9, carbohydrate antigen 19-9.

### 3.3 Predictive value of NPAR for postoperative prognosis in dCCA

**Figure 2:**
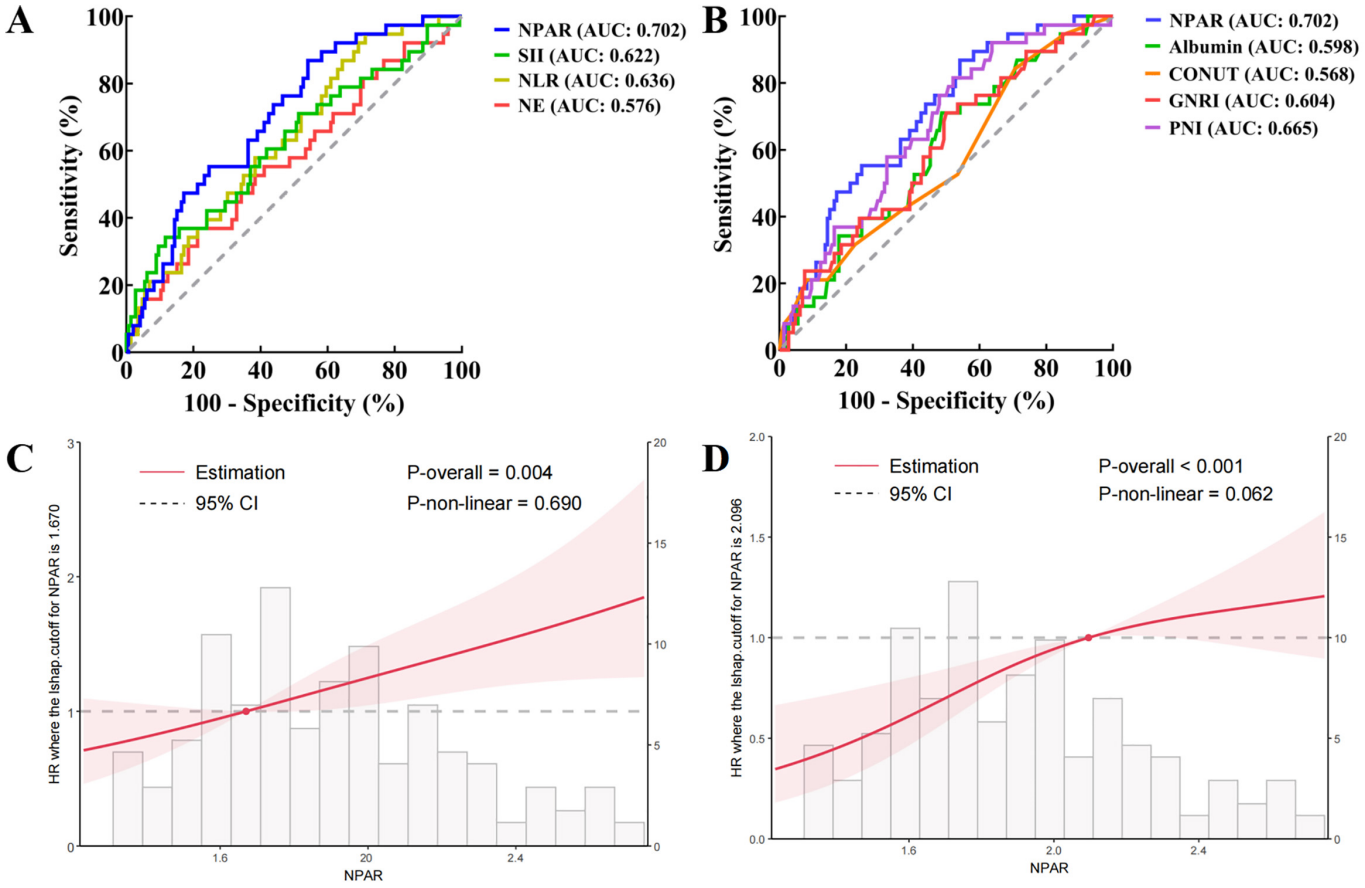
Prognostic value of preoperative neutrophil percentage-to-albumin ratio in distal cholangiocarcinoma patients: **(A)** comparison of receiver operator characteristic curve between neutrophil percentage-to-albumin ratio and neutrophil-derived indexes; **(B)** comparison of receiver operator characteristic curve between neutrophil percentage-to-albumin ratio and albumin-derived indexes; **(C)** restricted cubic splines of neutrophil percentage-to-albumin ratio and postoperative disease-free survival; **(D)** restricted cubic splines of neutrophil percentage-to-albumin ratio and postoperative overall survival (AUC, area under the receiver operator characteristic curve; NPAR, neutrophil percentage-to-albumin ratio; NLR, neutrophil-to-lymphocyte ratio; SII, systemic inflammatory index; NE, neutrophil; CONUT, controlling nutritional status; GNRI, geriatric nutritional risk index; PNI, prognostic nutritional index).

According to the ROC curve between preoperative NPAR and postoperative survival of dCCA patients, the AUC of NPAR in predicting postoperative prognosis was 0.702 (95% *CI*: 0.615–0.789). The VIF of absolute neutrophil count, neutrophil percentage, albumin and NPAR were 1.761, 2.083, 3.362 and 4.577 in multicollinearity test, indicating no severe multicollinearity among these indexes. This result confirmed that NPAR exerted independent prognostic value as a composite index rather than merely reflecting any individual component. To further evaluate the prognostic value of NPAR, we then compared the AUC of NPAR with other neutrophil-derived and albumin-derived indexes (Figures 2A, B). The result demonstrated that NPAR had superior prognostic value compared to other indexes. Further RCS analysis revealed a linear correlation between elevated NPAR and an increased risk of postoperative tumor recurrence and overall survival, consistent with our initial finding in univariate and multivariate analysis (Figures 2C, D). Thus, we believed that preoperative NPAR could serve as an effective prognostic index for dCCA.

**Figure 3:**
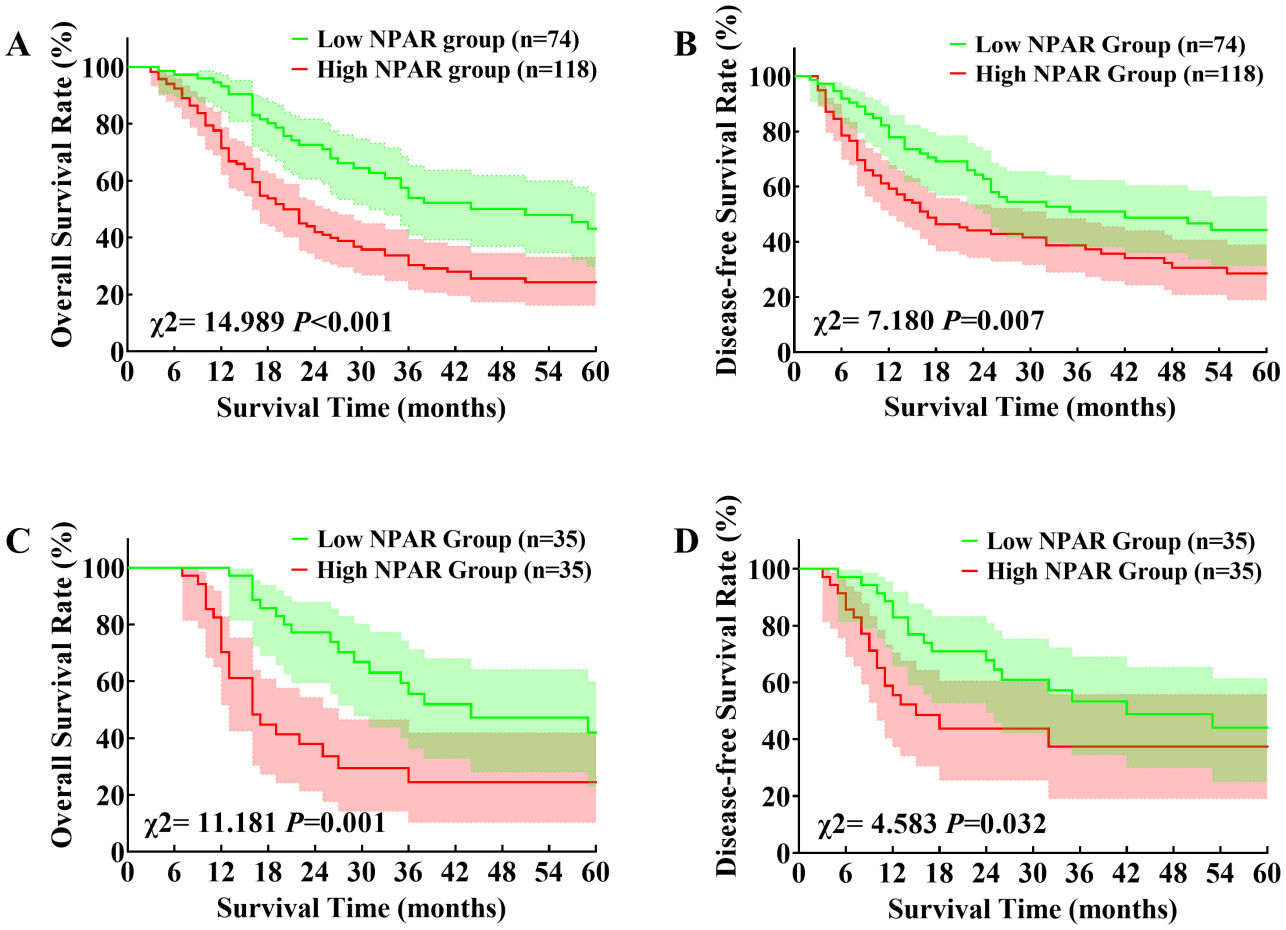
Comparison of overall survival and disease-free survival in low and high neutrophil percentage-to-albumin ratio group: **(A)** Kaplan–Meier curve of overall survival between low and high neutrophil percentage-to-albumin ratio group; **(B)** Kaplan–Meier curve of disease-free survival between low and high neutrophil percentage-to-albumin ratio group; **(C)** Kaplan–Meier curve of overall survival between low and high neutrophil percentage-to-albumin ratio group after propensity score matching; **(D)** Kaplan–Meier curve of disease-free survival between low and high neutrophil percentage-to-albumin ratio group after propensity score matching (NPAR, neutrophil-percentage-to-albumin ratio).

Furthermore, we determined the optimal cut-off value of NPAR in predicting postoperative survival of dCCA patients by ROC analysis. Its optimal cutoff value was 1.735, with a sensitivity of 86.8% and a specificity of 45.9%. Included patients were further divided into a low NPAR group (preoperative NPAR ≤ 1.735, *n* = 74) and a high NPAR group (preoperative NPAR > 1.735, *n* = 118) based on the optimal cut-off value. Intergroup comparison between low and high NPAR group was shown in Table 3, patients in high NPAR group had significantly higher preoperative absolute neutrophil count and total bilirubin, but lower preoperative albumin and prealbumin compared to those in the low NPAR group. The median OS time of patients in low and high NPAR group was 51 months and 20 months. The corresponding 1-, 3- and 5-year OS rate were 93.2%, 54.0%, 43.1% in low NPAR group, and 71.4%, 30.3%, 24.2% in high NPAR group, respectively (*P* < 0.001, Figure 3A). The median DFS time of patients in low and high NPAR group was 42 months and 17 months. The corresponding 1-, 3- and 5-year DFS rates were 77.9%, 50.9%, 44.3% in low NPAR group, and 59.2%, 38.7%, 28.6% in high NPAR group, respectively (*P* = 0.007, Figure 3B). After adjusting for confounding factors including preoperative absolute neutrophil count, total bilirubin, albumin and prealbumin with PSM, the median OS time (*P* = 0.001, Figure 3C) and DFS time (*P* = 0.032, Figure 3D) of patients in low NPAR group remained significantly longer than those in high NPAR group. These results collectively indicated that dCCA patients with preoperative NPAR > 1.735 tended to have significantly worse OS and DFS after surgery.

**Table 3:** Comparison of general condition between low and high preoperative neutrophil percentage-to-albumin ratio group.

**Variables**	**Pre-PSM**				**Post-PSM**			
	**Low NPAR group (*****n*** = **74)**	**High NPAR group (*****n*** = **118)**	**SMD**	***P*** **-value**	**Low NPAR group (*****n*** = **35)**	**High NPAR group (*****n*** = **35)**	**SMD**	***P*** **-value**
Gender (male/female)	43/31	73/45	0.077	0.604	19/16	20/15	0.058	0.810
Age (years)	63.50 (59.00, 70.00)	64.00 (61.00, 71.00)	0.084	0.226	64.00 (59.50, 71.00)	64.00 (61.00, 70.50)	−0.092	0.962
Smoking [case (%)]	25 (33.78)	32 (27.12)	−0.150	0.325	12 (34.29)	10 (28.57)	0.126	0.607
Preoperative biliary drainage [case (%)]	41 (55.41)	55 (46.61)	0.176	0.236	22 (62.86)	18 (51.43)	0.229	0.334
Preoperative WBC (× 10^9^/L)	6.00 (4.78, 7.10)	6.25 (5.18, 7.90)	0.263	0.110	6.60 (5.30, 7.40)	5.70 (5.10, 6.80)	−0.074	0.192
Preoperative NE (× 10^9^/L)	3.45 (2.49, 4.20)	4.47 (3.51, 5.75)	0.684	<0.001	3.88 (2.97, 4.78)	3.62 (3.25, 4.22)	−0.039	0.735
Preoperative ALB (g/L)	39.85 (36.85, 42.77)	33.95 (30.63, 36.67)	−1.182	<0.001	37.50 (35.35, 40.15)	36.10 (34.10, 38.85)	−0.250	0.207
Preoperative PAB (g/dl)	0.20 (0.15, 0.25)	0.14 (0.10, 0.19)	−0.756	<0.001	0.18 (0.14, 0.23)	0.16 (0.12, 0.22)	−0.240	0.317
Preoperative ALT (U/L)	56.00 (27.00, 150.50)	69.00 (41.00, 124.75)	−0.080	0.188	60.00 (26.50, 143.50)	60.00 (35.00, 130.00)	−0.081	0.833
Preoperative TB (μmol/L)	43.80 (19.35, 121.85)	138.10 (76.60, 226.10)	0.753	<0.001	77.40 (48.25, 141.75)	85.70 (54.05, 144.55)	−0.040	0.842
Preoperative CEA (ng/ml)	1.60 (1.10, 2.55)	2.00 (1.25, 3.00)	−0.156	0.108	1.80 (1.10, 2.95)	1.70 (1.15, 2.65)	−0.128	0.986
Preoperative CA19-9 (U/ml)	49.10 (18.38, 199.40)	74.40 (28.33, 311.10)	0.018	0.149	45.60 (16.85, 191.70)	81.70 (28.40, 516.65)	0.066	0.140
Operation time (hours)	10.00 (9.00, 11.00)	10.00 (8.00, 11.00)	−0.022	0.730	10.00 (9.00, 11.00)	10.00 (8.00, 12.00)	0.053	0.821
Intraoperative blood loss (ml)	400.00 (400.00, 600.00)	500.00 (400.00, 700.00)	0.068	0.493	500.00 (400.00, 800.00)	500.00 (400.00, 800.00)	0.161	0.678
TNM stage (I–II/III)	12/62	14/104	−0.135	0.391	3/32	5/30	−0.163	0.707
Tumor infiltraion (≤ 12mm/>12mm)	8/66	24/94	−0.237	0.085	3/32	8/27	−0.340	0.101
Lymph node metastasis [case (%)]	29 (39.19)	55 (46.61)	0.149	0.313	14 (40.00)	16 (45.71)	0.115	0.629
Portal vein invasion [case (%)]	8 (10.81)	18 (15.25)	0.124	0.381	3 (8.57)	7 (20.00)	0.286	0.172
Tumor differentiation (low/median-high)	21/53	45/73	0.201	0.166	9/26	13/22	0.237	0.303
Resection margin (R0/R1)	69/5	114/4	0.186	0.469	31/4	33/2	−0.246	0.669
Postoperative chemotherapy [case (%)]	39 (52.70)	57 (48.31)	−0.088	0.553	16 (45.71)	20 (57.14)	0.231	0.339
Postoperative hospital stay (d)	23.00 (17.00, 31.75)	21.50 (17.00, 32.00)	−0.013	0.700	22.00 (16.00, 34.00)	21.00 (16.50, 32.50)	−0.339	0.659
Postoperative complications [case (%)]	21 (28.38)	42 (35.59)	0.151	0.300	10 (28.57)	8 (22.86)	−0.136	0.584
Severe postoperative complications^*^ [case (%)]	10 (13.51)	16 (13.56)	0.001	0.469	5 (14.29)	2 (5.71)	−0.369	0.552
**Pancreatic fistula [case (%)]**								
Grade B fistula	7 (9.46)	21 (17.80)	0.218	0.111	3 (8.57)	5 (14.29)	0.163	0.707
Grade C fistula	3 (4.05)	6 (5.08)	0.047	1.000	1 (2.86)	0 (0.00)	−0.243	1.000
Biliary fistula [case (%)]	3 (4.05)	0 (0.00)	−0.331	0.108	3 (8.57)	0 (0.00)	−0.433	0.238
Delayed gastric emptying [case (%)]	9 (12.16)	12 (10.17)	−0.066	0.667	3 (8.57)	3 (8.57)	0.000	1.000
Abdominal infection [case (%)]	12 (16.22)	20 (16.95)	0.020	0.894	6 (17.14)	3 (8.57)	−0.306	0.475
Intra-abdominal bleeding [case (%)]	4 (5.41)	11 (9.32)	0.135	0.325	1 (2.86)	0 (0.00)	−0.243	1.000
Gastrointestinal bleeding [case (%)]	1 (1.35)	3 (2.54)	0.076	0.965	0 (0.00)	2 (5.71)	0.246	0.473
Re-operation [case (%)]	8 (10.81)	8 (6.78)	−0.160	0.325	5 (14.29)	2 (5.70)	−0.577	0.426

NPAR, neutrophil percentage-to-albumin ratio; WBC, white blood cell; NE, neutrophil; ALB, albumin; ALT, alanine transaminase; TB, total bilirubin; CEA, carcino-embryonic antigen; CA19-9, carbohydrate antigen 19-9; PSM, propensity score matching; SMD, standardized mean differences; ^*^Clavian–Dindo grade ≥ grade 3 was defined as severe postoperative complications.

### 3.4 Prognostic value of NPAR in dCCA patients at different stages

**Figure 4:**
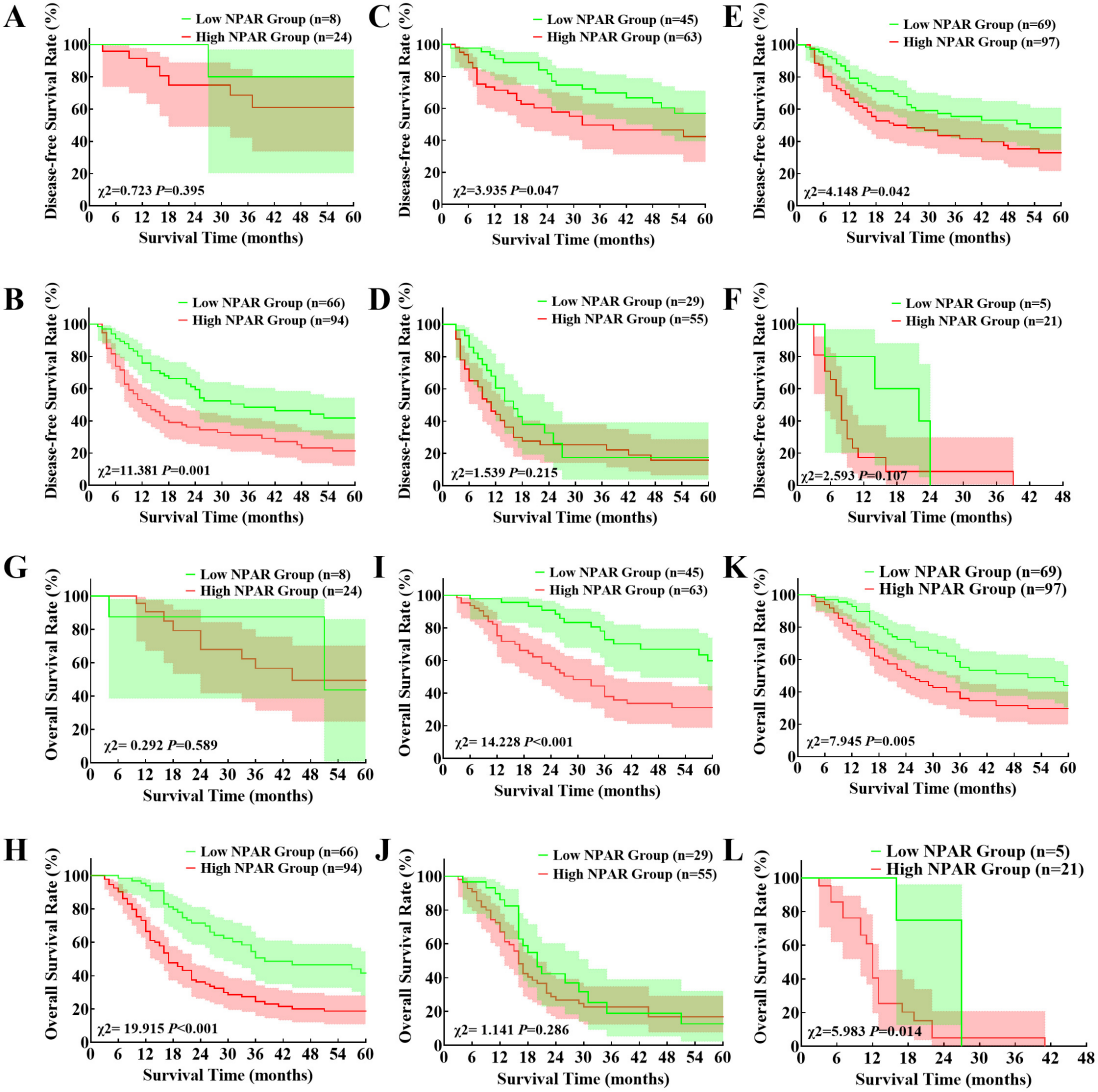
Comparison of disease-free survival and overall survival of low and high neutrophil percentage-to-albumin ratio group: **(A, B)** Kaplan–Meier curve of disease-free survival between low and high neutrophil percentage-to-albumin ratio group in patients with tumor infiltration depth ≤ 12mm **(A)** and tumor infiltration depth >12mm **(B)**; **(C, D)** Kaplan–Meier curve of disease-free survival between low and high neutrophil percentage-to-albumin ratio group in patients without **(C)** and with lymph node metastasis **(D)**; **(E, F)** Kaplan–Meier curve of disease-free survival between low and high neutrophil percentage-to-albumin ratio group in patients without **(E)** and with portal vein system invasion **(F)**; **(G, H)** Kaplan–Meier curve of overall survival between low and high neutrophil percentage-to-albumin ratio group in patients with tumor infiltration depth ≤ 12mm **(G)** and tumor infiltration depth >12mm **(H)**; **(I, J)** Kaplan–Meier curve of overall survival between low and high neutrophil percentage-to-albumin ratio group in patients without **(I)** and with lymph node metastasis **(J)**; **(K, L)** Kaplan–Meier curve of overall survival between low and high neutrophil percentage-to-albumin ratio group in patients without **(K)** and with portal vein system invasion **(L)**.

To assess the prognostic value of the NPAR cut-off value across various tumor stages, we performed a subgroup analysis in subgroups defined by tumor infiltration depth, portal vein system invasion, and lymph node metastasis. As shown in Figures 4A–F, the median DFS time was significantly longer in low NPAR group in patients with tumor infiltration depth >12mm, without portal vein invasion and without lymph node metastasis. This survival benefit was not observed in the other subgroups. The comparison of OS time in subgroups was shown in Figures 4G–L. Not only did low NPAR group have significantly longer postoperative OS time in these forementioned subgroups, but it was also associated with significantly improved OS in patients with portal vein system invasion.

### 3.5 Predictive value of model incorporating NPAR and clinical data in predicting postoperative prognosis of dCCA

**Figure 5:**
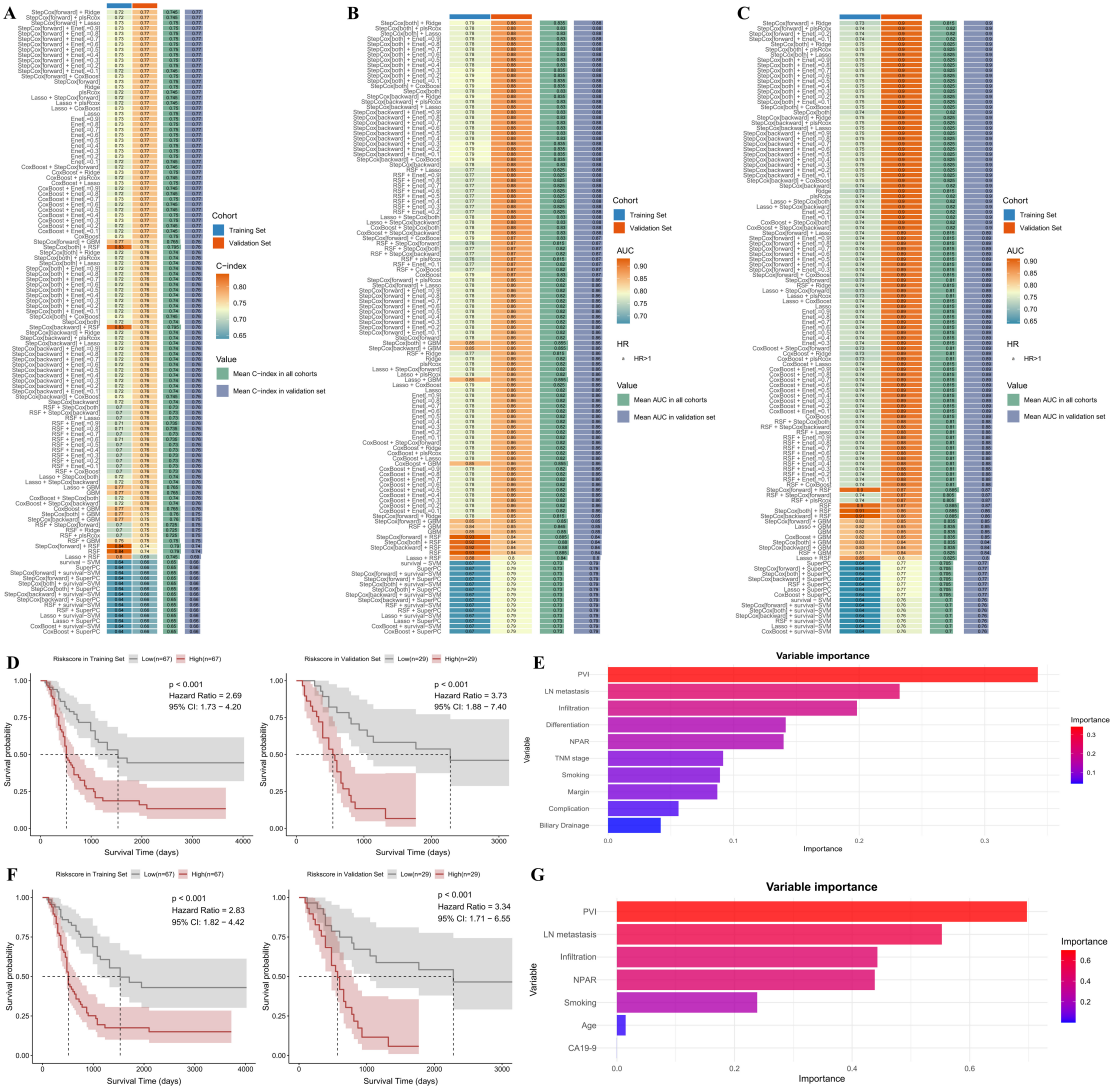
Selection and visualization of NPAR-based prediction model: **(A)** Heatmap of C-indexes of machine-learning models in predicting postoperative overall prognosis; **(B)** Heatmap of area under the receiver operator characteristics curve of different machine-learning models in predicting postoperative 3-year survival; **(C)** Heatmap of area under the receiver operator characteristics curve of different machine-learning models in predicting postoperative 5-year survival; **(D)** Kaplan–Meier curve of overall survival between low and high risk patients stratified by StepCox[forward] + Ridge model in training and validation set; **(E)** variable importance of StepCox[forward] + Ridge model; **(F)** Kaplan–Meier curve of overall survival between low and high risk patients stratified by StepCox[both] + Ridge model in training and validation set; **(G)** variable importance of StepCox[both] + Ridge model (CI, confidence interval; AUC, area under the curve; PVI, portal vein invasion; LN, lymph node; NPAR, neutrophil percentage-to-albumin ratio; CA19-9, carbohydrate antigen 19-9).

To improve the predictive accuracy of NPAR for postoperative prognosis in dCCA, we decided to develop a NPAR-based prediction model for postoperative prognosis in training set by utilizing a series of machine-learning models. In all included models, bidirectional stepwise Cox regression model (StepCox[both]) combined with Ridge regression exhibited the optimal predictive ability in predicting postoperative 3-year survival, while the stepwise forward Cox regression model (StepCox[forward]) combined with ridge Regression had the best predictive value for postoperative 5-year survival and the optimal C-index in predicting overall prognosis (Figures 5A–C). Both models could effectively predict postoperative overall prognosis according to Kaplan–Meier survival analysis (Figures 5D, F). After visualizing the variable importance of established models, preoperative NPAR served as an important factor in both models, confirming its prognostic value for postoperative overall survival in dCCA patients (Figures 5E, G). These results suggested that the prediction model incorporating NPAR can significantly improve the predictive value of NPAR in dCCA patients.

## 4 Discussion

Our study first reported the prognostic value of a novel inflammatory biomarker, NPAR, in predicting postoperative recurrence and long-term survival in a relatively large cohort dCCA patients. We further developed a model incorporating NPAR to predict postoperative prognosis of dCCA patients with good predictive ability. The findings of this research provided a novel indicator to improve the predictive accuracy of postoperative prognosis in dCCA patients. Moreover, since NPAR can be obtained from routine preoperative blood tests, it has the advantage of simple calculation and convenience for promotion. Therefore, the conclusion of this study has certain clinical value.

NPAR, a composite index including neutrophil percentage and albumin, can reflect the severity of systemic inflammation and has been utilized to predict the occurrence and prognosis of various tumors. According to Qu et al., patients with colorectal cancer had significantly higher NPAR level compared to those healthy controls, and elevated NPAR significantly increased the risk of developing colorectal cancer (17). In patients with oral cancer, Ko et al. found that patients with preoperative NPAR <16.93 had significantly longer postoperative DFS and OS time than those with NPAR ≥ 16.93, and confirmed high preoperative NPAR as an independent risk factor for postoperative tumor recurrence and overall prognosis (19). In a study including 213 patients with bladder cancer who received neoadjuvant therapy followed by radical surgeries, Ferro et al. discovered that patients with NPAR <18 had a significantly higher postoperative 5-year survival rate of 83% compared to those with NPAR ≥ 18 and identified higher NPAR as an independent risk factor for poor postoperative prognosis (18). In current research, we found that dCCA patients with high NPAR had worse DFS and OS time and served as an independent risk factor for poor postoperative outcomes, consistent with previous findings. However, regarding the optimal cut-off value of NPAR, the proposed cut-off value of 1.735 in our study differed from those found in previous research. This discrepancy may be attributed to variation in tumor types and the unit of serum albumin measurement utilized in different studies.

Inflammation is closely correlated to tumor development and progression and circulating neutrophils plays a pivotal role in this process. Szczerba et al. reported that circulating neutrophils can form CTC-NEUT clusters with tumor cells, thereby facilitating tumor metastasis. Furthermore, they observed that decreased neutrophils in circulation significantly delayed tumor metastasis and improved overall prognosis in murine model, highlighting the significant impact of neutrophils on cancer progression (20). Neutrophils can also protect tumor cells, foster vascularization, and promote tumor progression and metastasis by forming neutrophil extracellular traps, secreting exosomes rich in lncRNA and miRNA, and releasing cytokines such as vascular endothelial growth factor and matrix metalloproteinases-9 (21–23). Previous clinical studies have also reported a correlation between elevated absolute neutrophil counts and adverse outcomes in various types of cancer (24–26). In extrahepatic cholangiocarcinoma, Li et al. discovered that elevated preoperative absolute neutrophil count was an independent risk factor for poor postoperative prognosis and those dCCA patients with a neutrophil count > 3.68 × 10^9^/L had double risk of mortality, further validating the prognostic value of elevated neutrophil counts in cholangiocarcinoma (27). In the intergroup comparison of this study, we observed that patients with elevated NPAR had higher circulating neutrophil counts. Given that increased neutrophil counts is closely correlated with tumor progression and prognosis, we speculated that a higher absolute neutrophil count in high NPAR group may be a potential reason for the poor overall survival in these patients. Meanwhile, higher neutrophil percentage in these patients indicated a predominance of neutrophils in the circulating blood to some extent, which raised the risk of neutrophil-driven tumor progression and metastasis. Furthermore, an elevated neutrophil percentage also implies a decrease in absolute lymphocyte counts, indicating a weaker anti-tumor immune function. These may also correlate to the poorer postoperative outcomes in dCCA patients with high preoperative NPAR.

Serum albumin level, which was also included in NPAR, can reflect the patient nutritional status. For patients with cholangiocarcinoma, maintaining good nutritional status is crucial for reducing postoperative complications and improving overall survival. In a study including 140 patients with resectable dCCA, Okazoe et al. discovered that those diagnosed with malnutrition according to the GLIM criteria had a markedly decreased 5-year OS rate of only 29.3%, significantly lower than that of patients with normal nutritional status (28). Additionally, Rollins et al. found that unresectable cholangiocarcinoma patients who combined muscular steatosis secondary to malnutrition had significantly decreased OS time (29). Previous studies have confirmed that albumin, a marker of nutritional status, holds predictive value for postoperative outcomes in cholangiocarcinoma (30). Besides, composite indicators incorporating albumin such as the control nutritional status (COUNT) and PNI have been reported to serve as an effective prognostic value for dCCA patients, which may be associated with its ability to reflect the nutritional status of patients (12, 15). In our study, we found that patients in high NPAR group had lower serum albumin and prealbumin levels compared to those in low NPAR group, indicating a poorer nutritional status among the former. Based on this finding and the impact of nutritional status on dCCA prognosis, we hypothesized that poor nutritional status in high NPAR group might also be an important factor contributing to the worsened prognosis in high NPAR group. Nevertheless, the underlying mechanism by which NPAR influences the postoperative prognosis of dCCA patients remains to be elucidated in further researches.

Although significant differences in postoperative DFS and OS time were observed between low and high NPAR groups in all included patients, the intergroup comparison suggested significant differences in serum total bilirubin levels, absolute neutrophil counts and albumin level. According to previous studies, high serum bilirubin level, increased absolute neutrophil counts and low serum albumin level were independent risk factors for tumor recurrence and poor prognosis in patients with cholangiocarcinoma and were important confounding factors for our survival analysis (11, 27, 30, 31). Therefore, we applied PSM to adjust for these confounding factors and found that the OS and DFS time in low NPAR group remained better than that of high NPAR group. Multi-factor analysis also confirmed that the predictive value of NPAR was independent of these indicators, further verifying the clinical value of NPAR in predicting the postoperative prognosis of dCCA patients.

In current research we also categorized patients into different subgroups according to tumor infiltration depth, portal vein system invasion and lymph node metastasis to further explore the prognostic value of NPAR in different subgroups and guide its clinical application. Our study revealed that preoperative NPAR had prognostic value for postoperative tumor recurrence and overall survival in patients with tumor infiltration depth >12mm, without portal vein system invasion and lymph node metastasis. It could only predict overall prognosis in patients with portal vein system invasion, and had no predictive value in patients with tumor infiltration depth ≤ 12mm or combined lymph node metastasis. This result suggests that preoperative NPAR is of better predictive value in T3 stage dCCA with no lymph node metastasis. However, considering the relatively limited number of patients with stage T1–T2 tumors and portal vein system invasion in this study, we cannot definitively deny the predictive value of NPAR for these patients. Further research is necessary in future studies to clarify its potential role in this specific patient population.

In clinical practice, calculating the preoperative NPAR may assist in assessing the risk of postoperative mortality and recurrence, thereby facilitating risk stratification and assisting clinical decisions. Patients with preoperative NPAR ≤ 1.735 may better benefit from surgical treatment, therefore immediate surgery should be performed in these patients. For patients with preoperative NPAR > 1.735, clinicians should carefully evaluate the necessity of surgery and determine the optimal surgical strategy and timing. Besides, preoperative optimization strategies like preoperative nutritional support for these patients may also be considered in these patients to improve their clinical outcome. Also, a more rigorous and comprehensive postoperative follow-up protocol and earlier initiation of adjuvant chemotherapy may also be beneficial to those patients by enabling earlier detection of recurrence and timely intervention. However, since the predictive value of NPAR alone was not that satisfying, we built a machine-learning model incorporating it with other indexes and achieved satisfying prognostic value. This result underscores the necessity of using NPAR in combination with other prognostic markers, such as tumor markers, to enhance predictive performance when using in clinical practice.

This study also has some limitations. Firstly, as a single-center retrospective study, biases were inevitable in this study, thus its findings and conclusions should be verified through future multi-center, large-sample studies. Secondly, despite that the predictive value of predicting model incorporating NPAR was confirmed in our study, additional external validation is still necessary to further ascertain its predictive value. Thirdly, the relatively small number of patients with stage T1-2 tumors included in this study impacted the evaluation of predictive value of NPAR within this subgroup, making relevant findings less convincing and requiring subsequent researches. Fourth, our study is limited in its analysis of dCCA with portal vein invasion by a small subgroup sample size and potential temporal bias. Due to the extended study period, many patients from the earlier phase did not receive neoadjuvant therapy, which limits the generalizability of our findings to current clinical practice. These results therefore require cautious interpretation and future validation.

## 5 Conclusion

Preoperative NPAR has prognostic value in predicting postoperative tumor recurrence and long-term survival in dCCA patients. It exerts better prognostic ability in patients who had tumor infiltration depth >12mm, in the absence of portal vein system invasion and lymph node metastasis. A novel machine-learning model incorporating NPAR can further improve the predictive accuracy for postoperative prognosis in dCCA patients.

## Data Availability

The raw data supporting the conclusions of this article will be made available by the authors, without undue reservation.
